# Metastasectomy for Tumor-Infiltrating Lymphocytes: An Emerging Operative Indication in Surgical Oncology

**DOI:** 10.1245/s10434-017-6266-8

**Published:** 2017-11-29

**Authors:** Joseph G. Crompton, Nicholas Klemen, Udai S. Kammula

**Affiliations:** 1 Department of Surgery, University of California Los Angeles, Los Angeles, CA USA; 2 Department of Surgery, Yale University School of Medicine, New Haven, CT USA; 3 Division of Surgical Oncology, Department of Surgery, University of Pittsburgh School of Medicine, University of Pittsburgh Cancer Institute, Pittsburgh, PA USA; 4 UPMC Hillman Cancer Center, Pittsburgh, PA USA

## Abstract

Adoptive cell transfer (ACT) of tumor-infiltrating lymphocytes (TILs) is an emerging immunotherapy for metastatic cancer. Surgeons play a central role in ACT treatments by performing resection of tumors from which TILs are isolated. It is important that surgeons have familiarity with this emerging treatment method because it is increasingly performed for an expanding variety of solid tumors at institutions around the world. This report offers a brief introduction to ACT for cancer, highlights historical milestones in its development, and provides patient selection and operative considerations for surgeons called upon to perform metastasectomy for the purpose of isolating TILs.

## A Novel Way to Fight Cancer

In addition to the conventional methods of treating cancer (surgery, chemotherapy, and radiation), a treatment called adoptive cell transfer (ACT) is emerging that relies on the natural capacity of the immune system to target and eliminate tumor cells.^1^ The infiltration of tumor with immune cells, particularly T lymphocytes (also called tumor-infiltrating lymphocytes [TILs]), has long been observed, and recent findings have shown TILs to be predictive of patient survival in multiple solid tumor histologies.^2^–^5^ For example, the presence of T cells within metastatic tumors of colorectal origin can be a superior predictor of patient survival compared with the standard histopathologic methods currently used to stage colorectal cancer.^6^,^7^

**Fig. 1:**
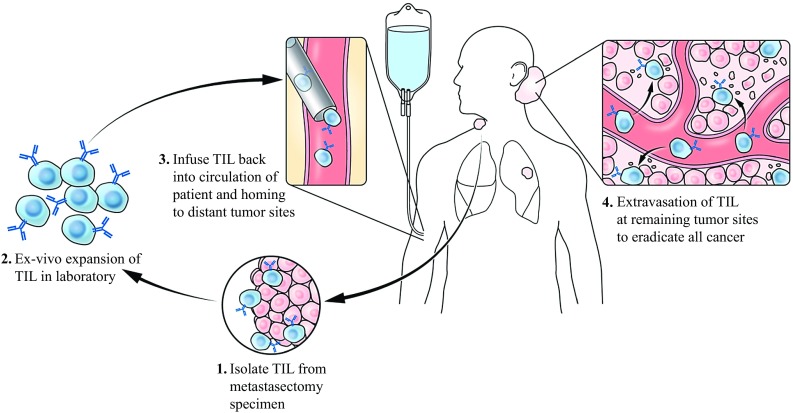
Adoptive cell transfer (ACT) of tumor-infiltrating lymphocytes (TILs). A generalized model of ACT using TILs is summarized with the following critical steps: (1) performance of metastasectomy to isolate TILs, (2) expansion of TILs in the laboratory with interleukin-2, (3) reinfusion of TILs after administration of lympho-depleting chemotherapy regimen (not shown), (4) TILs entering circulation and extravasating to tumor sites to eliminate malignant cells

Although T cells have a natural capacity to target tumors, it is thought that TILs become dysfunctional with time and lose their ability to eradicate tumor in late stages of cancer.^8^,^9^ To restore anti-tumor immunity, ACT first involves surgical resection of a tumor to isolate TILs from the tumor microenvironment. Next, TILs are expanded ex vivo with the T cell growth factor interleukin-2 (IL-2) 10,11 (Fig. 1). Before the TILs are adoptively transferred back into the patient, the existing immune system is transiently ablated with a lympho-depleting chemotherapy regimen that facilitates homeostatic responses favorable for engraftment and persistence of the transferred T cells. After transfer, TILs circulate throughout the body and traffic to distant tumor sites and tumor-draining lymph nodes, where they can mount an anti-cancer immune response.

With the exception of chemotherapy for germ cell tumors, few curative systemic therapies exist for adults with metastatic solid tumors.^12^ This has changed with the administration of immunotherapies such as high-dose-bolus interleukin-2, checkpoint blockade, and ACT.

In two recent studies of ACT using TILs, selected patients with advanced melanoma had a complete response rate of 22–24% and an overall response rate (measured by Response Evaluation Criteria in Solid Tumors [RECIST]) exceeding 50%.^13^,^14^ Importantly, more than 90% of the patients with a complete response to ACT remained disease free longer than 5 years.

In this brief review, we highlight the history of ACT for cancer and describe the central role of the surgeon, both in the development of this therapeutic approach and in its current practice. We also suggest practical guidelines that should be considered when a metastasectomy is performed to isolate TILs for the treatment of advanced cancer.

## A Brief History of Cell-Based Immunotherapy

Hence, it would seem fair to conclude that the lymphocyte is a necessary factor in cancer immunity

2 —James B. Murphy and John J. Morton (January 1915).

Historically, surgeon-immunologists such as John Hunter (who mapped the lymphatic system) and William Coley (who observed tumor regression in sarcoma patients with acute bacterial infections) have played pioneering roles in the development of immunotherapy for patients with cancer.^15^ In 1915, when immunology was still in its infancy, surgeon John J. Morton showed that rejection of tumors in mice was associated with a local infiltration of lymphocytes, a particularly prescient finding because he also showed that irradiation prevented lymphocytic infiltration, thereby enabling tumors to grow.^3^,^16^ In 1964, the British surgeon E. J. Delorme was the first to show that adoptive transfer of lymphocytes in a mouse model could inhibit carcinogen-induced sarcoma growth.^17^ Collectively, these findings helped give form to the notion that lymphocytes may be used therapeutically to treat patients with advanced cancer.

A breakthrough came in the 1970s with the discovery of interleukin-2, which enabled in vitro culture of T lymphocytes.^10^,^11^ This paved the way for the modern era of cellular immunotherapy, which began in 1988 when surgeon Steven A. Rosenberg and colleagues first demonstrated that ACT of TILs could effectively treat patients with advanced melanoma in studies conducted at the Surgery Branch of the National Cancer Institute.^18^ In these studies, 20 patients with metastatic melanoma were treated with ACT, and cancer regression was observed in diverse metastatic sites including liver, bone, and skin. Subsequent studies showed that the efficacy of ACT could be significantly improved when a lympho-depleting regimen of chemotherapy was administered before cell transfer.^19^,^20^

Murine models of immunosuppression before T cell transfer have suggested several benefits, including transient elimination of endogenous regulatory populations (e.g., regulatory T cells and myeloid derived suppressor cells) and immune cells that do not have specificity for cancer.^21^ Furthermore, lympho-depletion can augment availability of homeostatic cytokines and growth factors to enhance the engraftment and function of the transferred TILs. Although ACT was pioneered in patients with metastatic cutaneous melanoma, recent evidence shows that this treatment strategy can be extended to other cancers. Adoptive transfer of TILs in patients with advanced uveal melanoma, previously considered to be immunotherapy-resistant and in patients with human papillomavirus (HPV)-associated epithelial cancers (including cervical and oropharyngeal cancer) resulted in objective response rates of 35 and 33%, respectively.^22^,^23^ Case reports also show ACT resulting in partial responses of metastatic cholangiocarcinoma and colorectal cancer 24,25 after infusion of selected T cells that recognized the gene products of mutated cancer genes (termed neoantigens). Clinical trials of TIL therapy for patients with a variety of solid tumors are actively accruing (Table 1).

**Table 1:** Selected trials of adoptive cellular immunotherapy (ACT) with tumor-inflitrating lymphocytes (TILs) isolated by surgical metastasectomy^a^

Clinical trial	Summary	Cancer type(s)	Sponsor/collaborators
NCT01807182	ACT of TILs after combination chemotherapy	Melanoma	Fred Hutchinson Cancer Research Center; National Cancer Institute (NCI)
NCT02652455	ACT of TILs plus PD-1 blockade and CD137 agonism	Melanoma	H. Lee Moffitt Cancer Center and Research Institute; Bristol-Myers Squibb; Prometheus Inc.; Iovance Biotherapeutics, Inc.
NCT00604136	ACT of TILs	Melanoma	Hadassah Medical Organization
NCT02354690	ACT of TILs plus vemurafenib	Melanoma	Inge Marie Svane; Herlev Hospital
NCT02379195	ACT of TILs plus peginterferon	Melanoma	Inge Marie Svane; Herlev Hospital
NCT02926053	ACT of TILs	Renal cell carcinoma	Inge Marie Svane; Herlev Hospital
NCT02360579	ACT of TILs	Melanoma	Iovance Biotherapeutics, Inc.
NCT03083873	ACT of TILs	Squamous cell carcinoma of head and neck	Iovance Biotherapeutics, Inc.
NCT03108495	ACT of TILs	Cervical carcinoma	Iovance Biotherapeutics, Inc.
NCT01946373	ACT with or without dendritic cell vaccination	Melanoma	Karolinska University Hospital
NCT01955460	ACT with TGF-beta-resistant (DNRII) and NGFR-transduced T cells	Melanoma	M.D. Anderson Cancer Center; Cancer Prevention Research Institute of Texas
NCT01740557	ACT with T cells transduced with CXCR2 and NGFR	Melanoma	M.D. Anderson Cancer Center; National Cancer Institute (NIH/NCI); Prometheus Laboratories; Key Biologics, LLC
NCT00338377	ACT with or without dendritic cell immunization	Melanoma	M.D. Anderson Cancer Center; Prometheus Laboratories; Key Biologics, LLC; National Cancer Institute (NCI); Adelson Medical Research
NCT01174121	ACT of TILs	Gastrointestinal carcinoma, metastatic (colorectal, gastric, pancreatic, cholangio, hepatocellular)	National Cancer Institute (NCI); National Institutes of Health Clinical Center (CC)
NCT01993719	ACT of TILs	Melanoma	National Cancer Institute (NCI); National Institutes of Health Clinical Center (CC)
NCT02621021	Prospective randomized phase 2 trial of TILs plus IL-2, alone or after pembrolizumab	Melanoma	National Cancer Institute (NCI); National Institutes of Health Clinical Center (CC)
NCT02650986	ACT of TGFbDNRII-transduced TIL plus NY-ESO-1 reactive TCR transduced PBL	Solid tumors expressing NY-ESO-1	Roswell Park Cancer Institute; National Cancer Institute (NCI)
NCT03166397	ACT of TIL	Melanoma	Sheba Medical Center
NCT02421640	ACT of TIL following CCR	Nasopharyngeal carcinoma	Sun Yat-sen University
NCT02278887	ACT of TIL versus Ipilimumab	Melanoma	The Netherlands Cancer Institute; Copenhagen University Hospital at Herlev; University of Manchester
NCT01883297	ACT of re-stimulated TIL plus low-dose IL-2	Ovarian, fallopian or peritoneal cancer	University Health Network, Toronto
NCT01883323	ACT of TIL plus low-dose IL-2	Melanoma	University Health Network, Toronto
NCT02414945	ACT of TIL plus low-dose IL-2	Pleural mesothelioma	University Health Network, Toronto
NCT03158935	ACT of TIL followed by Pembrolizumab	Ovarian cancer; melanoma	University Health Network, Toronto; Merck Sharp & Dohme Corp.

*TGF* transforming growth factor, *NGFR* nerve growth factor receptor

^a^Additional details of each trial can be found by searching trial number at https://clinicaltrials.gov/

## Patient Selection for Act

Typically, patients being considered for ACT have often progressed through a number of conventional therapies and present with advanced-stage disease and limited life expectancy.^13^ As a part of a multidisciplinary team, surgeons play an integral role in selecting patients for ACT and planning a surgical approach with minimal morbidity, thereby ensuring that patients can be treated in a timely manner.

Eligible candidates for ACT should demonstrate adequate performance status (Eastern Cooperative Oncology Group [ECOG] 0 or 1). A commonly used lymphodepleting regimen includes cyclophosphamide (60 mg/kg) and fludarabine (25 mg/m^2^), which results in transient neutropenia for approximately 6–10 days, during which patients are vulnerable to septic complications (Table 2). Thus, treatment of patients at high risk for bacterial infections, such as those with biliary obstruction, chronic cholangitis, or indwelling biliary or ureteral stents, is relatively contraindicated. Lympho-depletion may further exacerbate chronic viral infections such as hepatitis C, hepatitis B, and human immunodeficiency virus. However, data are minimal on the outcomes for such patients after this type of preparative regimen. Transient thrombocytopenia also is common, so bleeding risk (e.g., from gastrointestinal or brain metastases) should be assessed before therapy.

**Table 2:** Patient selection for adoptive cell transfer (ACT) immunotherapy

Relative contraindications	Age < 18 or > 70 years
	ECOG performance status > 1
	Unacceptable risk of sepsis or bleeding during 7–10 days of neutropenia and thrombocytopenia
	Inability to tolerate interleukin-2 administration due to cardiopulmonary or renal insufficiency (some ACT protocols use low-dose or no IL-2)
	Current treatment with corticosteroids or immunosuppressive agents
Absolute contraindications	Primary immunodeficiency or chronic viral disease (e.g., HIV, HBV, HCV)
	Pregnancy
Other considerations	Large, symptomatic, or bleeding CNS lesions should be treated before ACT.
	Although trial eligibility may necessitate treatment with standard-of-care therapy before ACT, metastasectomy for TIL harvest can be performed first and the T cells frozen for later use.
	Patients in trials may require a radiographically evaluable target lesion for measurement of response to ACT.

*ECOG* Eastern Cooperative Oncology Group, *HIV* human immunodeficiency virus, *HBV* hepatitis B virus, *HCV* hepatitis C virus, *CNS* central nervous system, *TIL* tumor-infiltrating lymphocyte

Many ACT protocols administer high-dose-bolus intravenous IL-2 with cell transfer because it is thought to promote in vivo persistence and effector function of TILs. The administration of IL-2 can induce progressive capillary leak syndrome, which may cause respiratory compromise.^26^,^27^ Thus, eligible patients should have sufficient cardiopulmonary and renal reserve to tolerate the treatment.

Pregnancy is an absolute contraindication for ACT given the inherent risks to both the mother and the fetus. Small asymptomatic brain metastases are not a contraindication for ACT because durable regression of such lesions has been observed.^28^ However, symptomatic brain metastases, lesions larger than 1 cm, and edema or active bleeding should be managed before cell therapy to minimize neurologic complications. In the setting of primary autoimmune disorders requiring immunosuppression, ACT is contraindicated because of the suppressive effects on the transferred T cells (Table 2). Although checkpoint inhibitors (including anti-CTLA-4 and anti-PD-1 antibodies) have been associated with severe autoimmune side effects (including colitis, pneumonitis, and irreversible hypophysitis), these types of adverse events are rarely seen with TIL therapy.

**Fig. 2:**
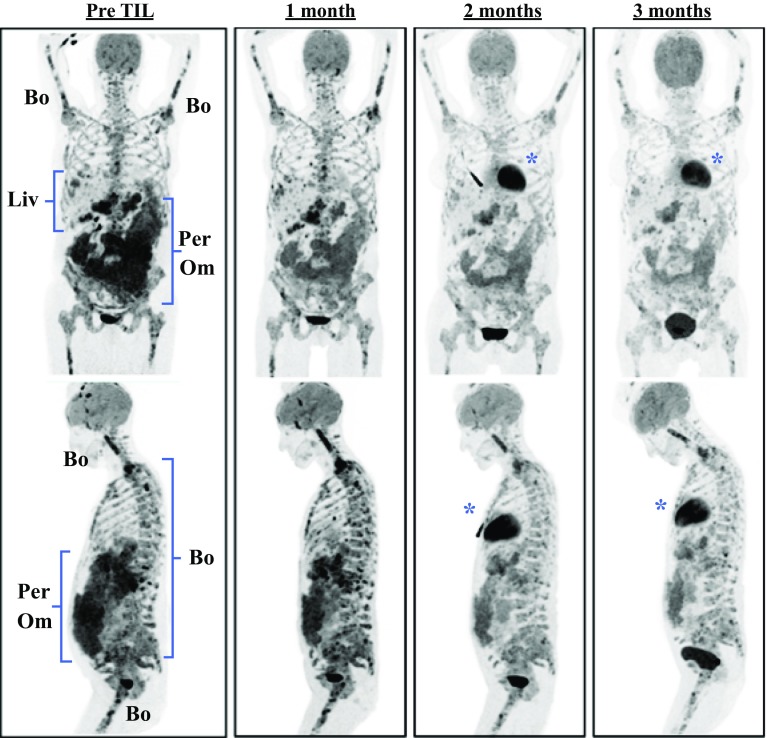
Regression of metastatic uveal melanoma after adoptive cell transfer. Example of rapid clinical response in a 52-year-old woman with metastatic uveal melanoma after adoptive cell transfer (ACT) of tumor-infiltrating lymphocytes (TILs). Pre-therapy and sequential post-therapy positron emission tomography (PET) scan images are shown, which demonstrate the partial regression by Response Evaluation Criteria in Solid Tumors (RECIST) of multiple peritoneal (Per), omental (Om), liver (Liv), and bone (Bo) metastases. *Normal physiologic 2-deoxy-2-[fluorine-18]fluoro-d-glucose (18F-FDG) uptake in the heart

Although no evidence exists to show that extensive tumor bulk directly impedes the efficacy of ACT, patients with extensive and rapidly progressive tumor burden often have limited time to wait for the manufacturing of a TIL product. Nonetheless, when appropriately timed, ACT can have a therapeutic impact on the most highly advanced patients. To illustrate this point, we present the case of a 52-year-old woman with significant metastatic burden from uveal melanoma, an orphan disease refractory to all conventional systemic treatments including immune checkpoint inhibitors.^23^ The patient presented with extensive bone, liver, peritoneal, and omental metastases, as demonstrated by positron emission tomography (PET) imaging (Fig. 2). After ACT using tumor reactive TILs, she demonstrated a dramatic and rapid regression of her disease burden associated with symptomatic relief from pain and early satiety.

## Operative Considerations for Til Metastasectomy

After patient selection, the next major decision facing the surgeon is selection of a tumor for TIL harvest (Table 3). Although tumor size does not correlate with efficacy of TIL therapy, tumors should be at least 2 cm in largest diameter to ensure an adequate quantity of tissue for successful processing. Larger tumors may have hypoxic and necrotic centers and do not necessarily yield higher quantities of TILs.

**Table 3:** Operative considerations in isolating tumor-infiltrating lymphocytes (TILs)

Consideration	Details
Tumor size	Tumor size does not correlate with TIL efficacy, but tumors should be at least 2 cm in largest diameter to obtain adequate yield of tissue for processing.
Irradiated tumors	Avoid harvesting TILs from a tumor site that has previously been irradiated.
Tumor site	Because TILs can be procured from a variety of tumor sites, favor surgical sites that result in minimal morbidity and consider laparoscopic approach.
Margins	Wide surgical margins and major organ resection are not typically necessary unless the tumor resection is being performed for curative intent. Avoid cutting through tumor to minimize risk of seeding tumor site.
Wound healing	Avoid harvest of superficial lesions if wound healing may be compromised.
Contamination	Ulcerating tumors and those with high suspicion for bacterial colonization can result in contamination of cultures. Isolation of TILs from bowel lesions is possible but may be associated with an increased risk of contamination.
Splenic lesions	Splenic tumors are not optimal for TILs because of theoretical concern that they may be enriched in bystander lymphocytes that are not tumor-reactive.
CNS lesions	Tumors metastatic to CNS have not been adequately assessed as a source of TILs for treatment.
Harvest	Refer to institutional guidelines for instructions on handling, processing, and labeling of tumor specimens.
Confirmation	Confirmation that the metastasectomy specimen contains malignant cells will ensure that benign or nodal tissue has not been inadvertently collected.

*CNS* central nervous system

No known correlation exists between the site of metastasectomy and the capacity to generate TILs. Cultures of TILs have been successfully initiated and expanded from diverse sites including liver, lymph node, lung, and gastrointestinal tract.^29^ Lymph nodes remain ideal sources of TILs because they can often be recovered with minimal postoperative morbidity. It should be cautioned that tumors procured from the lumen of the gastrointestinal (GI) tract or those that have occult colonization with bacteria or yeast often develop prohibitory contamination during ex vivo culture. An additional theoretical concern arises when TILs are isolated from lymphoid-rich tissues including the spleen or bowel (Table 3). Bystander T cells from these sites may expand ex vivo but will lack anti-tumor immune specificity. Thus, it is essential to confirm the presence and extent of tumor involvement when procuring lymphoid tissues.

Resection of visceral tumors can often be performed with a minimally invasive approach, as described in a series of 22 patients with stage M1c melanoma who underwent laparoscopic liver resection to procure tumor tissue for TIL generation.^30^ For many patients with widely metastatic disease, resection of metastases for TIL therapy does not demand wide surgical margins or major organ resection typically used for curative procedures (Table 3).

Once excised, tumors should be kept on ice and immediately transferred to a laboratory with personnel trained to dissect the tumor and initiate cultures. After isolation and expansion from fresh tumors, TILs in single-cell suspension can be cryopreserved for delayed treatment. As a final consideration, patients in clinical trials often require the presence of at least one evaluable target lesion so that the response to therapy can be measured by standard oncologic criteria, such as RECIST.

## Limitations of Act

Despite attempts to optimize patient selection, some patients may experience a decline in functional status after tumor procurement due to cancer progression or surgical complications to the point that they are no longer candidates for ACT. Furthermore, although experienced laboratories can routinely generate TIL cultures from metastatic tumors, in some circumstances, the harvested tumor fails to yield sufficient numbers of T cells for a treatment due to exhaustion of the T cells, lack of tumor reactivity, or culture contamination.^31^ Accumulation of these risks implies that a proportion of patients will undergo TIL harvest but ultimately will not be eligible for ACT.^32^ Patients should be counseled regarding this possibility.

## Future Research Directions

In analyses of cutaneous melanoma patients who achieved durable and complete clinical responses after ACT, the infused TILs were found to immunologically recognize one or more neoantigens, the gene products of somatic mutations.^33^ These findings provide compelling evidence that tumor-specific mutations can generate neo-epitopes that elicit robust autologous immunologic responses in cutaneous melanoma patients. In fact, the high mutational burden in cutaneous melanoma, largely driven by ultraviolet mutagenesis, may explain the susceptibility of this cancer to a variety of immunotherapeutic approaches. Consequently, other highly mutated cancers including non-small cell lung cancer, bladder cancers, and microsatellite-unstable GI cancers may represent additional cancers to be investigated with TIL therapy. It should be noted that these histologies have demonstrated favorable responses to immunotherapy using checkpoint inhibitors.^34^,^35^

In an effort to target cancers with a low mutational burden, it may be possible to select or purify low-frequency T cell populations that possess neoantigen reactivity.^24^,^25^,^36^ Furthermore, preclinical data suggest that the efficacy of ACT is correlated with the differentiation status or “stemness” of transferred T cells.^37^,^38^ Methods to minimize differentiation during ex vivo expansion, manipulate T cell functional avidity, and enhance the metabolic fitness of T cells may further improve clinical outcomes.^39^–^42^ Finally, combinatorial treatments that couple ACT with immune-modulating agents, such as checkpoint inhibitors, also may improve efficacy.^43^

## Conclusions

Adoptive cell transfer has shown early therapeutic promise for a variety of advanced solid cancers. Although initially limited to a few institutions, recent innovations have increased the feasibility and effectiveness of this treatment method, and it currently is offered at multiple cancer centers around the world. Treatment with ACT remains a relatively resource-intensive and costly therapy that requires large-scale institutional support and a full good manufacturing practice (GMP) laboratory. The challenge of making ACT more widely available may involve either commercialization or designated centers that can provide consulting, manufacturing, and regulatory support. Surgeons have played a pioneering role in the development of immunotherapy for cancer. Through their direct involvement with patients, the ability to procure tumors, and their unique insights into cancer biology, surgeons will continue to make important contributions that increase the safety and effectiveness of cell-based therapies.
